# Database of persistent organic pollutants in umbilical cord blood: Concentration of organochlorine pesticides, PCBs, BDEs and polycyclic aromatic hydrocarbons

**DOI:** 10.1016/j.dib.2019.104918

**Published:** 2019-11-30

**Authors:** Raúl Cabrera-Rodríguez, Octavio P. Luzardo, Maira Almeida-González, Luis D. Boada, Manuel Zumbado, Luis Alberto Henríquez-Hernández

**Affiliations:** a Toxicology Unit, Research Institute of Biomedical and Health Sciences (IUIBS), Universidad de Las Palmas de Gran Canaria, Paseo Blas Cabrera s/n, 35016, Las Palmas de Gran Canaria, Spain; b Spanish Biomedical Research Center in Physiopathology of Obesity and Nutrition (CIBERObn), Spain

**Keywords:** Persistent organic pollutant, Organochlorine pesticides, Polychlorinated biphenyls, Bromodiphenyl ethers, Polycyclic aromatic hydrocarbons, Newborn, Umbilical cord blood

## Abstract

Persistent organic pollutants (POPs) have been banned over the last decades for being damaged to the environment and to the health of humans and animals. However, due to their lipophilic nature and resistance to degradation, they are frequently detected in biological samples. Its presence has been associated with the increased risk of suffering from different diseases in human series, being newborns and children especially sensitive. The present data reports umbilical cord blood levels of twenty organochlorine pesticides (aldrin, dieldrin, endrin, o,p'-DDD, p,p'-DDD, o,p'-DDE, p,p'-DDE, o,p'-DDT, p,p'-DDT, endosulfan alfa, endosulfan beta, endosulfan sulphate, heptachlor, HCB, αHCH, βHCH, δHCH, lindane, methoxychlor and mirex), eighteen polychlorinated biphenyls (congeners 28, 52, 77, 81, 101, 105, 114, 118, 123, 126, 138, 153, 156, 157, 167, 169, 180 and 189), eight bromodiphenyl ethers (congeners 28, 47, 85, 99, 100, 153, 154 and 183), and sixteen polycyclic aromatic hydrocarbons (acenaphthalene, acenaphthene, anthracene, benzo(a)anthracene, benzo(a)pyrene, benzo(b)fluoranthene, benzo(g,h,i)perylene, benzo(k)fluoranthene, chrysene, dibenzo(a,h)anthracene, fluoranthene, fluorine, indene(1,2,3-cd)pyrene, naphthalene, phenanthrene and pyrene). A total of 447 samples, representing 86.6% of the total births during the recruited period (March 1, 2015, to April 30, 2016), were available for POP analyses. POPs were determined in a Gas Chromatography (GC) system equipped with an automated sampler (Models 7890B and 7693; Agilent Technologies, Palo Alto, CA, USA) for gas chromatographic separations. The detection of the analytes was performed using a Triple Quad 7010 mass spectrometer (Agilent Technologies). All of the measurements were performed as triplicate measurements, and the means were used for the calculations. Data are reported in ng/mL. The present data also includes birth parameters, including weight, length, cranial perimeter, Apgar score and congenital malformations, and data referred to mothers (harmful habits, chronic diseases, and anthropometric/demographic characteristics).

**Table:** Specifications Table

Subject	Toxicology
Specific subject area	Detection and quantification of persistent organic pollutants in umbilical cord blood from 447 newborns from La Palma (Canary Islands, Spain)
Type of data	Table
How data were acquired	Gas chromatography - mass spectrometry Instruments: GC model 7890B and Triple Quad 7010, respectively. Agilent Technologies, Palo Alto, CA, USA.
Data format	Raw
Parameters for data collection	Birth weight, length, and cranial perimeter of newborns were recorded at delivery. Data on congenital malformations were detected at birth, identified and recorded. Gestational age was calculated based on the last menstrual period. Other anthropometric and biological characteristics of the mother included age, parity, type of delivery, and previous miscarriages.
Description of data collection	Cord blood samples were collected in EDTA tubes after collection by venipuncture of the umbilical cords obtained immediately after delivery. Samples were stored at −80 °C until the moment of their processing for analysis. Recruited period: March 1, 2015 to April 30, 2016. All samples were recruited in La Palma (Canary Islands)
Data source location	Institution: Toxicology Unit, Clinical Sciences Department, Universidad de Las Palmas de Gran Canaria City/Town/Region: Las Palmas de Gran Canaria (Gran Canaria, Canary Islands) Country: Spain
Data accessibility	With the article
Related research article	Cabrera-Rodríguez R, Luzardo OP, Almeida-González M, Boada LD, Zumbado M, Acosta-Dacal A, Rial-Berriel C, Henríquez-Hernández LA. Association between prenatal exposure to multiple persistent organic pollutants (POPs) and growth indicators in newborns. Environmental Research 2019 Apr; 171:285–292. https://doi.org/10.1016/j.envres.2018.12.064.

**Table:** 

**Value of the Data** • The present data is useful because reports levels of 62 POPs in a series of 447 umbilical cord blood samples, together with the demographic and clinical parameters recorded for the series. The present data help interpreting effects caused by the inadvertent exposure to these hazardous compounds. • These data will benefit everyone conducting biomonitoring studies in human populations, especially those who conduct meta-analysis. • Data provided can be reanalysed, compared to other datasets in other series, and used for meta-analyses. • Although many studies have been published reporting levels of POPs in human populations, few include such amount of substances in a population as sensitive as newborns. • The present series includes 87% of the births registered in La Palma Island (Canary Islands, Spain) during 2016, which allows having a biomonitorization that includes almost all of a population segment in a specific period of time.

## 1 Data description

The data contains umbilical cord blood concentration of organochlorine pesticides, PCBs, BDEs and polycyclic aromatic hydrocarbons. Data are provided in excel format containing the following data (Table in Supplementary data):

• Demographical and clinical data referred to the mothers: age at birth (years), type of delivery (vaginal/caesarean), nulliparity (yes/no), lactation (months), miscarriages (yes/no), diseases — including diabetes, arterial hypertension and hypothyroidism (yes/no), smoking habit (yes/no)
• Clinical data referred to the newborn: gestational age (weeks), sex (male/female), birth weight (g), length (cm), head circumference (cm), malformations at birth (yes/not), Apgar score
• Concentration of pollutants in ng/mL: organochlorine pesticides (aldrin, dieldrin, endrin, o,p'-DDD, p,p'-DDD, o,p'-DDE, p,p'-DDE, o,p'-DDT, p,p'-DDT, endosulfan alfa, endosulfan beta, endosulfan sulphate, heptachlor, HCB, αHCH, βHCH, δHCH, lindane, methoxychlor and mirex), eighteen polychlorinated biphenyls (congeners 28, 52, 77, 81, 101, 105, 114, 118, 123, 126, 138, 153, 156, 157, 167, 169, 180 and 189), eight bromodiphenyl ethers (congeners 28, 47, 85, 99, 100, 153, 154 and 183), and sixteen polycyclic aromatic hydrocarbons (acenaphthalene, acenaphthene, anthracene, benzo(a)anthracene, benzo(a)pyrene, benzo(b)fluoranthene, benzo(g,h,i)perylene, benzo(k)fluoranthene, chrysene, dibenzo(a,h)anthracene, fluoranthene, fluorine, indene(1,2,3-cd)pyrene, naphthalene, phenanthrene and pyrene). A summary table reporting the ranges of concentration of the pollutants is showed in Table 1.

**Table 1:** Ranges of concentration of the POPs (ng/mL).

OCPs	Range	PAHs	Range	PCBs	Range	BDEs	Range
Aldrin	0.002–0.161	Acenaphthalene	0.001–0.231	PCB 101	0.002–0.269	BDE 100	0.046–0.051
o,p'-DDD	ND	Acenaphthene	0.007–0.306	PCB 105	0.007–0.062	BDE 153	0.026–0.067
p,p'-DDD	0.011–0.095	Anthracene	0.181–0.181	PCB 114	0.024–0.037	BDE 154	0.006–0.072
o,p'-DDE	0.068–0.068	Benzo(a)anthracene	0.193–0.229	PCB 118	0.001–0.151	BDE 183	0.072–0.078
p,p'-DDE	0.001–3.762	Benzo(a)pyrene	0.160–0.164	PCB 123	0.002–0.189	BDE 28	0.050–0.075
o,p'-DDT	0.010–0.040	Benzo(b)fluoranthene	0.053–0.162	PCB 126	0.001–0.084	BDE 47	0.014–0.137
p,p'-DDT	0.095–1.027	Benzo(g,h,i)perylene	0.052–0.475	PCB 138	0.001–0.220	BDE 85	ND
Dieldrin	0.001–0.723	Benzo(k)fluoranthene	0.001–0.232	PCB 153	0.002–0.234	BDE 99	0.039–0.056
Endosulfan alfa	0.066–0.069	Chrysene	0.093–0.122	PCB 156	0.025–0.025		
Endosulfan beta	0.035–0.097	Dibenzo(a,h)anthracene	0.019–0.338	PCB 157	ND		
Endosulfan sulphate	0.003–0.055	Fluoranthene	0.001–1.382	PCB 167	0.002–0.029		
Endrin	ND	Fluorene	0.001–1.828	PCB 169	0.017–0.023		
Heptachlor	0.023–0.026	Indene(1,2,3-c,d)pyrene	0.081–0.088	PCB 180	0.005–0.143		
HCB	0.001–0.350	Naphthalene	0.023–22.196	PCB 189	0.079–0.081		
α-HCH	ND	Phenanthrene	0.005–8.417	PCB 28	0.001–2.129		
β-HCH	0.010–1.340	Pyrene	0.004–1.187	PCB 52	0.002–0.354		
δ-HCH	0.023–0.028			PCB 77	0.003–0.060		
Lindane	0.056–1.351			PCB 81	0.013–0.091		
Methoxychlor	0.004–0.132						
Mirex	0.019–0.09						

*Abbreviations:* POPs, persistent organic pollutants; OCP, organochlorine pesticides; PAHs, polycyclic aromatic hydrocarbons; PCBs, polychlorinated biphenyls; BDE, bromodiphenyl ethers; ND, non detected.

## 2 Experimental design, materials, and methods

All samples were recruited in the island of La Palma (Canary Islands, Spain). The present series represents the 86.6% of the total births during the recruited period (March 1, 2015, to April 30, 2016). Birth parameters were recorded at the delivery room, as previously reported [1]. Data referred to mothers were recorded and included anonymously in the database. Both parents were required to sign an informed consent. This study was approved by the Ethics Committees of the Hospital of La Palma and the University of Las Palmas de Gran Canaria in accordance with the Declaration of Helsinki. The samples were stored according to the regulations dictated by the Spanish Law of Biomedical Investigation of 2007 (Law 14/2007) and the data were saved according to the Data Protection Act (Ley Orgánica 15/1999).

Sample preparation, instrumentation, and quality assurance/quality control are extensively exposed in a previous publication [2,3]. We used PASW Statistics version 19.0 (SPSS Inc., Chicago, IL, USA) to manage the database and perform statistics [2].

## Conflict of Interest

The authors declare that they have no known competing financial interests or personal relationships that could have appeared to influence the work reported in this paper.
